# The Effect of Different Chemical Surface Treatments on the Bond Strength of Resin-Matrix Ceramic Repaired with Resin Composite

**DOI:** 10.1055/s-0044-1785531

**Published:** 2024-05-14

**Authors:** Satit Prabriputaloong, Nantawan Krajangta, Awiruth Klaisiri

**Affiliations:** 1 Division of Restorative Dentistry, Faculty of Dentistry, Thammasat University, Pathum Thani, Thailand

**Keywords:** adhesive agent, bond strength, surface treatment, universal adhesive

## Abstract

**Objective** This study investigates the effect of different chemical surface treatment protocols with different functional monomers of universal adhesives on the shear bond strength between resin-matrix ceramic and resin composite.

**Materials and Methods** Eighty resin-matrix ceramics (Shofu block HC) were built and designed into eight groups of ten specimens and surface treated with HC primer (HC) and/or three universal adhesives (single bond universal [SBU], Scotchbond universal plus [SBP], and Tetric N-bond universal [TNU]) assigning follows; group 1, nonsurface treated; group 2, HC; group 3, SBU; group 4, HC + SBU; group 5, SBP; group 6, HC + SBP; group 7, TNU; group 8, HC + TNU. A template was put on the specimen center, and then pushed packable resin composite. Mechanical testing machinery was used to examine the samples' shear bond strength (SBS) values. To examine failure patterns, the debonded specimen surfaces were examined by a stereomicroscope.

**Statistical Analysis** The one-way analysis of variance method was used to evaluate the data, and the Tukey's test was used to determine the significant level ( *p* < 0.05).

**Results** The highest SBS was obtained in group 6 (39.25 ± 1.65 MPa). Group 1 (4.15 ± 0.54 MPa) had the lowest SBS. Group 6 exhibited the highest percentage of cohesive failure patterns (70%). High SBS values were frequently correlated with the surface treatment groups and the cohesive failure patterns.

**Conclusion** The application of HC primer prior to the universal adhesive is an alternative protocol for enhancing the repair bond strength between resin-matrix ceramic and resin composite interfaces. Moreover, the application of HC primer prior to the SBP is the best strategy for resin-matrix ceramic and resin composite repairs.

## Introduction

Nowadays, there is a large quantity of dental material products currently available, such as computer-aided design/computer-aided manufacturing (CAD/CAM) resin composite blocks and dental ceramics. A system of classification would be helpful in giving useful information regarding the material's use (e.g., anterior or posterior teeth), the kind of restoration that works best for it (partial or full coverage), and bonding techniques (adhesive). 1 Three groups were distinguished for ceramic restorative materials. The presence of specific qualities can influence a product's composition. This includes polycrystalline ceramics, glass-matrix ceramics, and resin-matrix ceramics. 1

Direct resin composite fillings are often utilized and can yield acceptable and dependable esthetic outcomes for both anterior and posterior fillings; nevertheless, numerous disadvantages have been documented, including inadequate mechanical characteristics and unpredictable color stability. 2 In comparison with resin-based composites used in direct fillings, indirect restorations are distinguished by greater mechanical qualities and are more stable in color stability, particularly because extraoral curing can achieve a higher degree of conversion. 3 High-pressure and/or high-temperature polymerization are utilized in standardized industrial manufacturing methods for CAD/CAM resin composite blocks. These can enhance polymer cross-linking and the characteristics of the material. Another benefit of CAD/CAM resin composite blocks is that they can be finished more quickly than ceramics because they do not require a firing procedure after milling. In addition, they are simple to polish, finish, and repair. 4 In contrast to other ceramic systems, resin-matrix ceramic material has lower rates of fracture propagation, inert biaxial flexural strength, and a low elastic modulus. 5 6 Hence, clinical fractures could happen. Sometimes resin composite is used to repair fractures, 7 but it is important to consider the fracture's size, location, and urgency for a new restoration. The benefits of repairing methods include time and resource savings, a decrease in microbial adherence to the fracture, and the preservation of the tooth remnant. 8

In recent years, the manufacturer of Shofu block HC, which is one type of resin-matrix ceramic, has been introduced. Shofu block HC is a type of dental restorative material that is used to create tooth-colored inlays, onlays, and crowns. It is made from a resin-matrix ceramic material that contains both glass fillers and a resin matrix, which give it strength and durability. Shofu block HC is known for its high translucency and excellent shade matching capabilities. Shofu block HC is a reliable and versatile restorative material that can be used for a variety of dental purposes. In recent times, a new resin primer (HC primer, Shofu, Kyoto, Japan) including methyl methacrylate (MMA) was launched. Hagino et al found that sandblasting and the use of HC primer for surface treatment of Shofu block HC improved stronger bonding between Shofu block HC and resin composite. 9 However, the surface treatment of Shofu block HC with different functional monomers of universal adhesive has not yet been reported.

*In vitro* research points to the use of different functional monomers of universal adhesive as one surface treatment that may enhance the bond ability between resin-matrix ceramic (Shofu block HC) and resin composite repairs. The objective of this study was to explore the chemical surface treatment protocols with different functional monomers of universal adhesives for resin-matrix ceramics (Shofu block HC) repaired with resin composites. The null hypothesis was that the chemical surface treatment protocols with different functional monomers of universal adhesives for resin-matrix ceramics (Shofu block HC) repaired with resin composites do not differ in each protocol.

## Materials and Methods

### Preparation of Resin-Matrix Ceramic Specimens

**Fig. 1:**
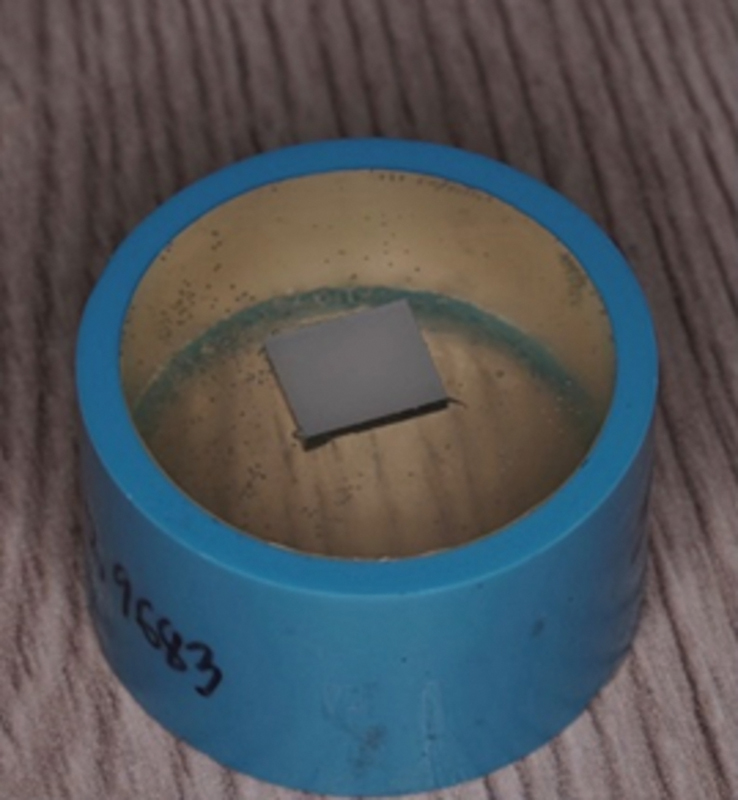
The aged resin-matrix ceramic was inserted in a polyvinyl chloride tube.

Eighty pieces of Shofu Block HC (Shofu, Kyoto, Japan) were cut in a rectangle using a microcutting instrument (Accuton-50 wafer cutting instrument, Struers, Ohio, United States) with a 6 × 7 mm size and a 1.5 mm thickness. The resin-matrix ceramic specimens were aged by the thermocycling machine (Proto-tech, Microforce, Oregon, United States) with 5,000 thermal cycles between 5 and 55°C with 30-second dwell time and 5 seconds of transfer time. 10 The resin-matrix ceramic specimens were placed within an epoxy resin-filled polyvinyl chloride tube ( Fig. 1 ). To standardize the surface roughness of the resin-matrix ceramic surfaces, 3M abrasive sheet (3M, Minnesota, United States) was used to sand them using a 600-grit silicon carbide sandpaper. All of the samples were given a 10-minute immersion in distilled water using an ultrasonic cleaning.

The resin materials that were used in the present investigation are described in Table 1 .

**Table 1:** The resin materials that were utilized for this research

Materials	Compositions
Shofu Block HC (Shofu, Kyoto, Japan); Lot: 0721594	UDMA, TEGDMA, filler; silica powder, micro fumed silica, zirconium silicate, 61% by weight
HC Primer (Shofu, Kyoto, Japan); Lot: 072109	10–20% MMA, 10–20% acetone, UDMA, polymerization initiator and others
Singlebond universal (3M, Neuss, Germany); Lot: 9720542	10-MDP, Bis-GMA, HEMA, DMA, methacrylate functional copolymer, silane, filler, initiators, ethanol, water
Scotchbond universal plus (3M, Neuss, Germany); Lot: 9527356	HEMA, 2-propenoic acid, 2-methyl-, diesters with 4,6-dibromo-1,3-benzenediol 2-(2-hydroxyethoxy)ethyl 3-hydroxypropyl diethers, 2-propenoic acid, 2-methyl-, reaction products with 1,10-decanediol and phosphorus oxide, 2-propenoic acid, 2-methyl-, 3(triethoxysilyl)propylester, reaction products with silica and 3(triethoxysilyl)-1-propanamine, synthetic amorphous silica, fumed, crystalline-free, ethanol, water, (3-aminopropyl)triethoxysilane, camphorquinone, N,N-dimethylbenzocaine, methacrylic acid, Acetic acid, copper(2 + ) salt, monohydrate
Tetric N-bond universal (Ivoclar Vivadent, Schaan, Liechtenstein); Lot: Z04RG6	Bis-GMA, HEMA, UDMA, 10-MDP, ethanol, diphenyl (2,4,6-trimethylbenzoyl) phosphine oxide
Resin composite (Harmonize A2E shade, Kerr Corporation, California, United States); Lot: 8690666	Bis-GMA, TEGDMA, EBPADMA, zirconia/silica cluster filler (2–3 µm) comprised of 20 nm spherical fumed silica and 5 nm zirconia particles, prepolymerized filler

**Abbreviations:** Bis-GMA, bisphenol A-glycidyl methacrylate; DMA, dimethacrylate; EBPADMA, Ethoxylated bisphenol A dimethacrylate; HEMA, 2-hydroxyethyl methacrylate; MMA, methyl methacrylate; 10-MDP, 10-methacryloyloxydecyl dihydrogen phosphate; TEGDMA, triethylene glycol dimethacrylate; UDMA, urethane dimethacrylate.

### Sandblast Technique

**Fig. 2:**
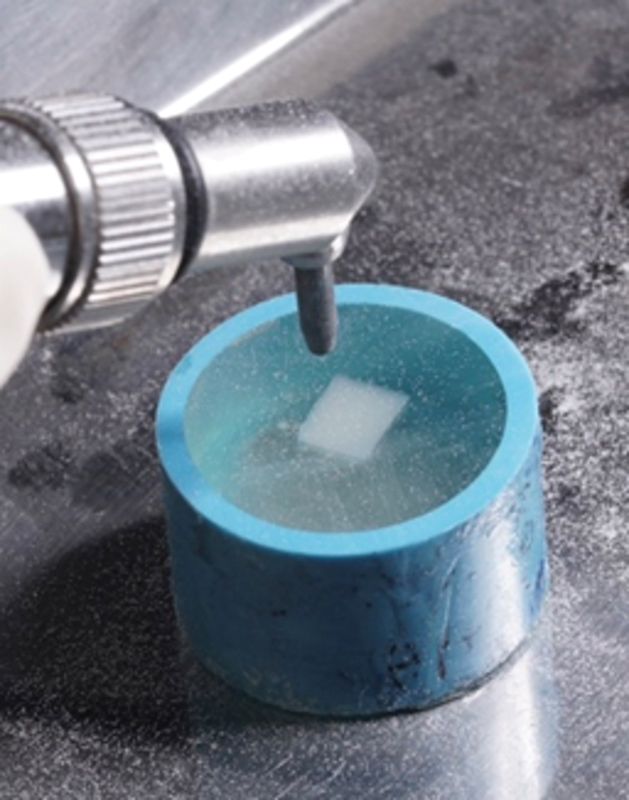
The aged resin-matrix ceramic was sandblasted.

The specimens were sandblasted by 50-micron Al _2_ O _3_ particles positioned 10 mm apart for 10 seconds at a pressure of 2 bars ( Fig. 2 ). 10 After sandblasting, the specimens were cleaned and allowed to air dry for 10 seconds employing a triple syringe.

### HC Primer Surface Treatment

The specimen's surface received a 20-second microbrush treatment for the HC primer, and the excess primer was cleaned up with a new microbrush and allowed to air dry for approximately 20 seconds. Then it was light-cured with a light-emitting diode curing equipment (Elipar, 3M ESPE, St. Paul, Minnesota, United States) for 10 seconds as per the manufacturer's recommendation. If the HC primer was applied before the universal adhesive application, it was not light-cured.

### Universal Adhesive Surface Treatment

The universal adhesive was treated with the microbrush to the surface of the specimen for 20 seconds, and the excess universal adhesive was cleaned up by a new microbrush. The solvent of the universal adhesive was removed by gently allowing it to air dry for about 5 seconds. It was allowed to air dry until the surface became shiny and there was no more liquid movement. After that, it received a 20-second light cure.

### Resin Composite Application

The resin-matrix ceramic surface-treated specimens were assigned at random to eight groups ( *n* = 10 per group) and surface treated with HC primer (HC) and/or three universal adhesives (single bond universal [SBU], Scotchbond universal plus [SBP], and Tetric N-bond universal [TNU]) assigning follows;

Group 1: nonsurface treated with chemical agents
Group 2: surface-treated with HC; (HC)
Group 3: surface-treated with SBU; (SBU)
Group 4: surface-treated with HC prior to application to SBU; (HC + SBU)
Group 5: surface-treated with SBP; (SBP)
Group 6: surface-treated with HC prior to application to SBP; (HC + SBP)
Group 7: surface-treated with TNU; (TNU)
Group 8: surface-treated with HC prior to application to TNU; (HC + TNU)

On the surface-treated specimen center, an ultradent mold measuring 2.0 mm in thickness and 2.0 mm in diameter was placed. The A2E shade packable resin composite (Harmonize, Kerr Corporation, California, United States) was pressed and light-polymerized for 40 seconds. An ultradent mold was pulled out, and then light-polymerized for 40 seconds again. All of the samples underwent a 1-day incubation procedure in an incubator (Contherm 160M, Contherm Scientific Ltd., Lower Hutt, New Zealand) with distilled water at 37 degrees Celsius.

### Shear Bond Strength (SBS) Determination and Fracture Mode Inspection

**Fig. 3:**
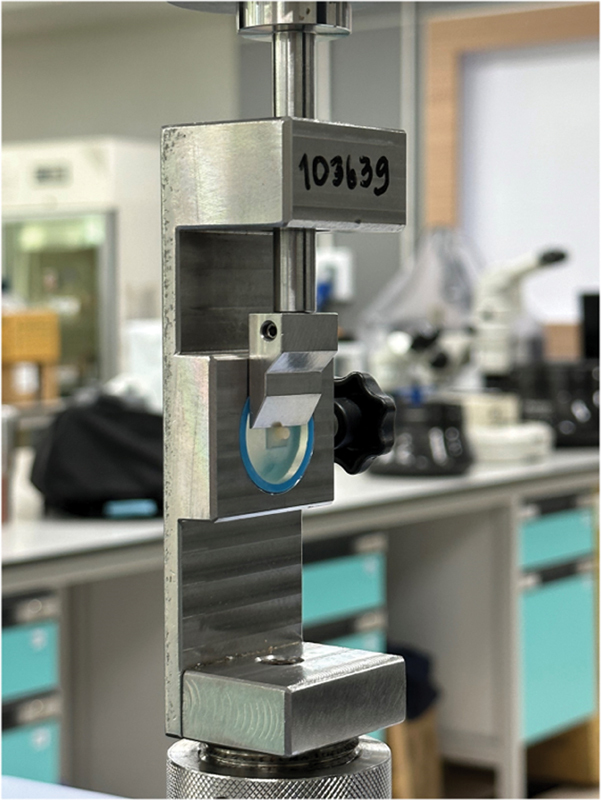
The shear bond strength analysis.

The SBS values were evaluated at a test speed of 0.5 mm per minute using a universal testing system (AGS-X 500N, Shimadzu Corporation, Kyoto, Japan; Fig. 3 ). The adhesion zone and the bond breakdown force were divided to determine the SBS value.

The fracture mode patterns of resin-matrix ceramic and resin composites were inspected by a stereomicroscope at x50 magnification. To categorize the fracture modes, three patterns were employed 11 12 13 14 (i) an adhesive pattern (fracture on the interface between resin-matrix ceramic and resin composite), (ii) a cohesive pattern (fracture in resin-matrix ceramic or resin composite), and (iii) a mixed pattern (combined adhesive and cohesive failure patterns).

### Data Analysis

The data were investigated employing a one-way analysis of variance, with a significance level of *p* -value less than 0.05 established by the Tukey's test.

## Results

In Table 2 , the mean SBS values and standard deviation are shown. The highest SBS values were displayed in group 6 (39.25 ± 1.65 MPa). Group 1 found the significantly lowest SBS value (4.15 ± 0.54 MPa). The bond strength values of group 2 (26.77 ± 3.43 MPa) and group 3 (27.50 ± 2.31 MPa) were not significantly different when used compared to group 7 (27.77 ± 2.40 MPa). Moreover, the SBS values of group 4 (33.74 ± 1.01 MPa) and group 5 (33.24 ± 3.66 MPa) were not significantly different from group 8 (32.74 ± 1.64 MPa).

**Table 2:** The mean SBS ± SD and percentage of failure mode pattern

Groups	Mean SBS ± SD	Percentage of failure mode		
		Adhesive	Mixed	Cohesive
1. No treatment	4.15 ± 0.54 ^a^	100	0	0
2. HC	26.77 ± 3.43 ^b^	20	60	20
3. SBU	27.50 ± 2.31 ^b^	20	60	20
4. HC + SBU	33.74 ± 1.01 ^c^	0	40	60
5. SBP	33.24 ± 3.66 ^c^	0	40	60
6. HC + SBP	39.25 ± 1.65 ^d^	0	30	70
7. TNU	27.77 ± 2.40 ^b^	10	60	30
8. HC + TNU	32.74 ± 1.64 ^c^	0	40	60

Abbreviations: SBP, Scotchbond universal plus; SBS, shear bond strength; SBU, single bond universal; SD, standard deviation; TNU, Tetric N-bond universal.

The value with identical letters indicates no statistically significant difference.

Table 2 provides an overview of the failure mode distribution pattern. After being fractured, all fractured specimens in group 1 were identified as having adhesive failure mode. Additionally, in groups 2 to 8, mixed and cohesive failure modes were raised. Group 6 exhibited the highest percentage of cohesive failure patterns (70%).

**Fig. 4:**
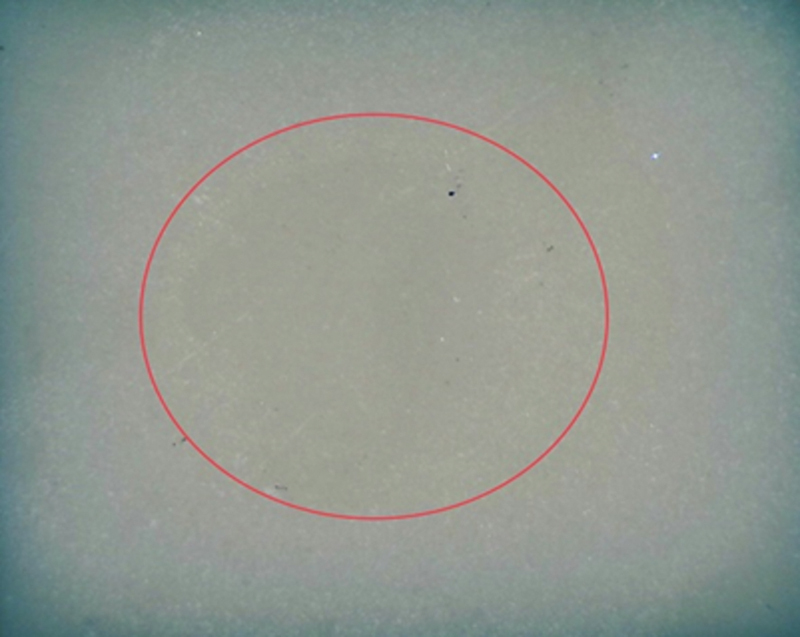
The stereomicroscope picture showing all adhesive failures in group 1.

**Fig. 5:**
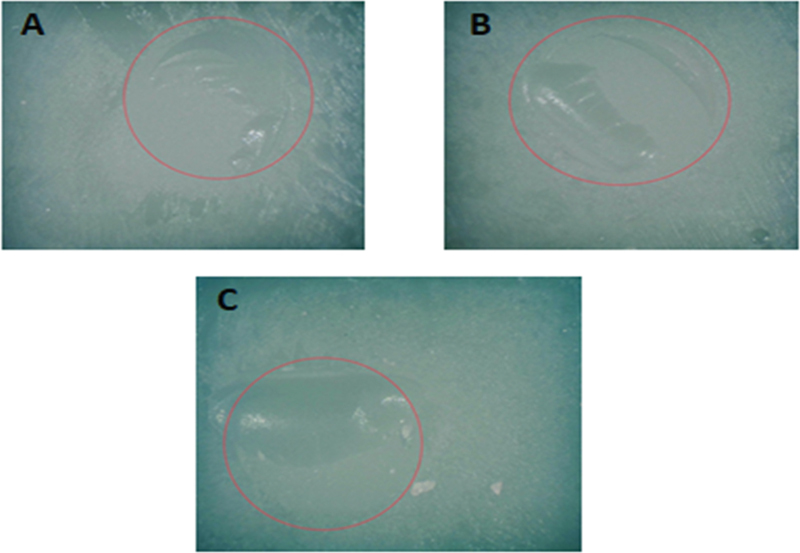
The stereomicroscope pictures showing the most mixed failures in groups 2, 3, and 7: ( **A** ) group 2; ( **B** ) group 3; ( **C** ) group 7.

**Fig. 6:**
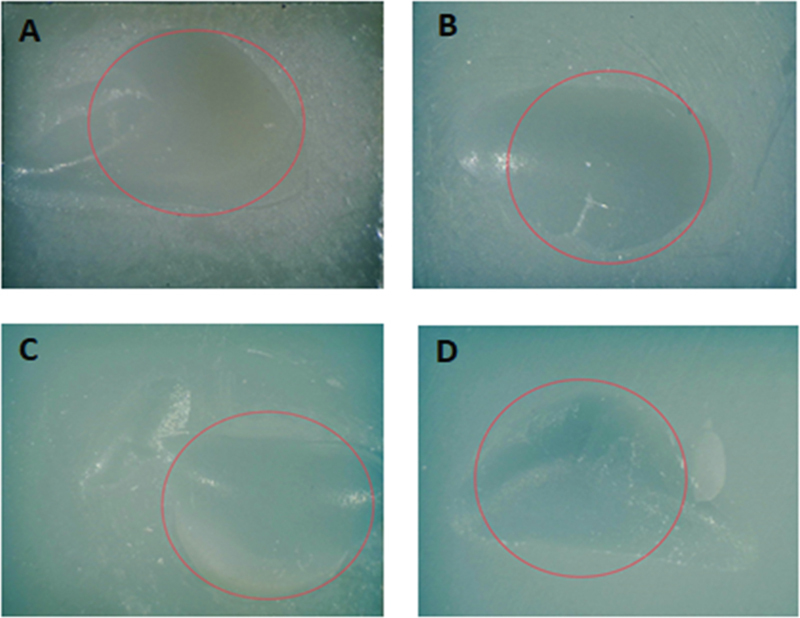
The stereomicroscope pictures showing the most cohesive failures in resin-matrix ceramic in groups 4, 5, 6, and 8: ( **A** ) group 4; ( **B** ) group 5; ( **C** ) group 6; ( **D** ) group 8.

In the part of stereomicroscope analysis, the stereomicroscope pictures of examples of the highest percentage failure mode in each group (adhesive, mixed, and cohesive fracture modes) are demonstrated in Figs. 4 5 6 . Group 1 demonstrated the highest percentage of adhesive failure patterns ( Fig. 4 ). Groups 2, 3, and 7 exhibited a high percentage of mixed failure patterns ( Fig. 5 ). Meanwhile, groups 4, 5, 6, and 8 presented a high percentage of cohesive failure patterns ( Fig. 6 ).

## Discussion

This study investigated the chemical surface treatment protocols with different functional monomers of universal adhesives for resin-matrix ceramics (Shofu block HC) repaired with resin composites. The outcomes show that there is significant variation in the SBS values for each group. Consequently, the null hypothesis was disproved.

To establish a strong bond between resin composites and resin-matrix ceramic materials, it is crucial to understand how various surface alterations affect the way these materials interact. 10 15 To achieve clinical efficacy, the resin-matrix ceramic and resin composite have to be attached to one another in a powerful and durable way. To enhance mechanical bonding, the resin-matrix ceramic surface roughness must be caused by sandblasting and acid etching. 15 The sandblasting technique enhanced the bond strength values as compared with no surface treatment. 10 15 To improve chemical adhesion between the resin-matrix ceramic and resin composite, use a chemical agent and/or adhesive agent to condition the resin-matrix ceramic surface. This is an efficient method. 9 10 16 Numerous micromechanical surface alteration methods as well as chemical surface treatment using chemical agents and/or adhesive systems have been proposed as ways to enhance the resin-matrix ceramic and resin composite's potential for repair bonding. 9 10 15 16

According to the micromechanical retention, sandblasting protocol significantly improved the bond ability between resin-matrix ceramic and resin composite. 15 The air abrasion goal is to improve the material's surface roughness by generating matrix irregularities and enhancing surface energy. 16 17 This also results in improved anchoring via microretention by increasing the resin composite wettability. 18 Other benefits of sandblasting a CAD/CAM resin include cleansing the bonding area following saliva contamination by presenting a freshly cleaned area. 19 Furthermore, an enhancement in SBS between resin-matrix ceramic and resin composites by using MMA monomers and/or adhesive agents could be created. 9 10 16

In our outcomes, the resin-matrix ceramic surface treated with HC primer (26.77 ± 3.43 MPa) exhibited significantly improved SBS compared to the nonsurface-treated group (4.15 ± 0.54 MPa). The interaction between resin-matrix ceramic and resin composites was stronger, which may be due to the high-thickness bonding interface from the HC primer. 9 The main compositions of HC primer are MMA and urethane dimethacrylate (UDMA), which can form a thick film of resin material between resin-matrix ceramic and resin composite interfaces. This layer may absorb the polymerization stress and reduce the stress concentration between the interfaces. 9 Due to the presence of the low molecular weight monomers UDMA and MMA in the HC primer tested in this study, it is possible that the primer moved into the resin-matrix ceramic's sandblasted surface treatment and then cured there. This is in agreement with a prior study 9 that found greater SBS after using HC primer compared to specimens that were not surface-treated. Moreover, the MMA in the HC primer could swell the matrix of resin-matrix ceramic; subsequently, the UDMA monomer could penetrate into the matrix of resin-matrix ceramic 20 ; as a result, it has a higher SBS than the nonsurface-treated group.

For resin-matrix ceramic surfaces-treated, earlier investigations found that silane, a universal primer (which also contains 10-MDP and silane), or a universal adhesive produced superior results. 15 21 22 The bonding of the silane to the SiO _2_ unprotected fillers or 10-MDP to zirconia unprotected fillers in resin-matrix ceramics may be related to predicted advances for universal primers or universal adhesives. 15 Previous studies reported the beneficial effects of universal adhesive containing silane after sandblasting for the surface treatment of resin-matrix ceramic. 10 23 On the contrary, Yao et al discovered that the reaction of self-condensation of the silane agents might result in a weak bonding capacity in universal adhesives containing silane with low pH values. 24 Some authors indicated that a universal adhesive containing silane has improved or decreased the bond strength associated with the type of silane in the universal adhesive. 25 26 In this study, the SBU group (27.50 ± 2.31 MPa) has a significantly lower SBS compared to the SBP group (33.24 ± 3.66 MPa). The silane agent is present in both SBU and SBP. The SBU contains only 3-methacryloxypropyltrimethoxysilane (3-MPTS), but the SBP contains mixed silane agents, which are composed of 3-methacryloxypropyltriethoxysilane (3-MPTES) and 3-(aminopropyl)triethoxysilane (APTES). Leelaponglit et al 26 found that the silane agent in SBP has a significantly positive effect compared with SBU. The 3-MPTS in SBU is nonstable in the universal adhesive's mildly acidic self-etch, resulting in a silane agent that is not effective. 27 28 On the contrary, the mixed silane agents in SBP can be effective in universal adhesives at low pH, which protects them from cyclic self-condensation and promotes the silane agent's ability to bond to the silica filler in resin-matrix ceramic; the causation of the shear bond ability of the SBP is higher than the SBU.

For the universal adhesives containing 10-MDP (SBU, SBP, and TNU), the SBS of these groups (SBU; 27.50 ± 2.31 MPa and TNU; 27.77 ± 2.40 MPa) was not significantly different from the HC primer-treated group (26.77 ± 3.43 MPa), except for the SBP group (33.24 ± 3.66 MPa), where the SBS was higher than the HC primer-treated group. The SBU is a combination made from 10-MDP monomer and 3-MPTS silane coupling agent, of which 3-MPTS is not effective. 26 The TNU is composed of only 10-MDP monomers. The 10-MPD is composed of the phosphate functional monomer, which can chemically direct bond to the zirconium oxide, 12 which is why it might chemically adhere to the zirconium filler in resin-matrix ceramic. For these reasons, the SBS of the SBU and the TNU was not significantly different from the HC primer-treated group. Moreover, the use of HC primer before the universal adhesive application raised the bond ability more than the HC primer or universal adhesive treated alone. The three possible mechanisms are (i) The HC primer may reduce and absorb the polymerization stress at the interface between the resin composite and resin-matrix ceramic 9 (ii) The 10-MDP monomer in universal adhesive can also chemically direct adhesion to the zirconium filler in resin-matrix ceramic; (iii) The MMA and UDMA monomers in the HC primer may be copolymerized with a universal adhesive agent monomer, which forms interpenetrating polymer connections. 29 30 For these reasons, the combination of HC primer applied to prior universal adhesive has significantly improved the SBS more than the HC primer or universal adhesive treated only.

As a result, the HC + SBP group (39.25 ± 1.65 MPa) has the highest SBS compared to all groups. Because the SBP consists of 10-MDP and mixed silane (3-MPTES and APTES). The 10-MDP might chemically adhere to the zirconium filler, and the mixed silane can have a positive effect on the silica filler in resin-matrix ceramic. 26 When the resin-matrix ceramic was surface-treated with the combination of HC primer and SBP, the HC primer, 10-MDP, and mixed silane all effectively action bonded to the resin-matrix ceramic. The four potential methods are as follows: (i) The HC primer could reduce and absorb the stress resulting from polymerization, and could swell the resin-matrix ceramic resin matrix; (ii) The universal adhesive agent monomers may be copolymerized with the MMA and UDMA monomers in HC primer; (iii) The universal adhesive's 10-MDP monomer has the ability to chemically assist adherence to the resin-matrix ceramic's zirconium filler; (iv) Chemical bonds between the mixed silane and the silica filler in resin-matrix ceramics can be formed successfully.

Three patterns are used as a classification system for the fracture failure types in certain features of the shown failure mode 11 12 13 14 : (i) a pattern of adhesive failure that appears when the resin-matrix ceramic and resin composite are broken at the interface; (ii) a cohesive failure mode inside the resin-matrix ceramic that breaks; and (iii) a mixed failure mode that results from combining cohesive and adhesive failure modes. In this investigation, adhesive failure was found in all specimens in group 1 ( Fig. 4 ). Moreover, mixed ( Fig. 5 ), and cohesive failure modes became more prevalent in groups 2 through 8. Cohesive failure modes in groups 4, 5, 6, and 8 ( Fig. 6 ) were often associated with elevated SBS. There was a definite relationship between bond ability and the overall number of cohesive patterns; the quantity of cohesive modes rose as bond ability increased. 31 32 33 If the material bond strength value is closer to its cohesive fracture mode, the repair will be more successful. 34

In the context of this research's clinical application, an alternate protocol in clinical dental practice is the application of HC primer prior to the universal adhesive, which is the best strategy for enhancing the repair bond strength between resin-matrix ceramic and resin composite. This protocol effectively enhanced the bond strength of resin-matrix ceramic (Shofu block HC) and resin composite repairs.

The design of this research study was limited since it could not be transferred to other resin-matrix ceramics because it emphasized the use of a single resin-matrix ceramic (Shofu block HC). The resin-matrix ceramic and resin composites' SBSs could only be ascertained by the incubated specimen 24 hours after bonding. Thermocycling might be used in the future to assess the longevity of repairs made of resin composites and resin-matrix ceramic materials. The effectiveness of an adhesion approach in a clinical setting is associated with a number of factors, including the SBS. It is crucial to thoroughly examine the findings of our investigation as a result.

## Conclusion

The current *in vitro* investigation's results, within the limitations of the study, demonstrated the advantages of HC primers and universal adhesive agents in encouraging chemical bonding between resin-matrix ceramics (Shofu block HC) repaired with resin composites. The application of HC primer prior to the universal adhesive is an alternative protocol for enhancing the repair bond strength between resin-matrix ceramic and resin composite interfaces. Moreover, the application of HC primer prior to the SBP is the best strategy for resin-matrix ceramic and resin composite repairs.
